# SUMO2 deficiency impairs endogenous regulation of oxidative stress and apoptosis in myocardial ischemia-reperfusion injury

**DOI:** 10.3389/fcell.2026.1842772

**Published:** 2026-08-04

**Authors:** Xiuying Zhang, Wei Zhao, Jia Zhao, Ni Fan, Yu Wang, Aimin Xu, Wei Yang, Jianhui Rong

**Affiliations:** 1 School of Chinese Medicine, Li Ka Shing Faculty of Medicine, University of Hong Kong, Pokfulam, Hong Kong, China; 2 School of Basic Medical Sciences, Guizhou Medical University, Guiyang, China; 3 The University of Hong Kong Shenzhen Institute of Research and Innovation (HKU-SIRI), 5/F, Key Laboratory Platform Building, Shenzhen Virtual University Park, Shenzhen, China; 4 Zhujiang Hospital, Southern Medical University, Guangzhou, Guangdong Province, China; 5 Department of Pharmacology and Pharmacy, Li Ka Shing Faculty of Medicine, University of Hong Kong, Pokfulam, Hong Kong, China; 6 Department of Medicine, Li Ka Shing Faculty of Medicine, University of Hong Kong, Pokfulam, Hong Kong, China; 7 Department of Anesthesiology, Duke University Medical Center, Durham, NC, United States

**Keywords:** cardioprotection, myocardial ischemia-reperfusion injury, p53, SUMO2, sumoylation

## Abstract

**Introduction:**

Myocardial ischemia-reperfusion injury (MIRI) is a major obstacle in the treatment of myocardial infarction and is closely associated with oxidative stress and cardiomyocyte death. Although endogenous protective mechanisms are activated during ischemic stress, they are often insufficient to prevent injury. SUMOylation is an important post-translational modification involved in cellular stress adaptation, and SUMO2 may contribute to the endogenous cardioprotective response.

**Methods:**

To investigate the role of SUMO2 in MIRI, we generated heart-specific SUMO2 knockout (SUMO2-KO) mice by crossing SUMO2-loxP mice with Myh6-Cre mice and assessed cardiac injury after myocardial infarction. In parallel, adeno-associated virus-mediated SUMO2 overexpression was used to assess the protective effect of SUMO2. In H9c2 cells subjected to oxygen-glucose deprivation, SUMO2 silencing was performed to examine reactive oxygen species (ROS) production, Bax/Bcl-2 balance, apoptosis, and p53-associated signaling.

**Results:**

SUMO2-KO mice exhibited more severe myocardial injury and worse cardiac dysfunction after ischemic injury. In contrast, SUMO2 overexpression markedly attenuated cardiac damage. In H9c2 cells, SUMO2 silencing increased ROS production, disrupted the Bax/Bcl-2 balance, and enhanced apoptosis. These effects were accompanied by alterations in p53-associated signaling.

**Discussion:**

These findings suggest that SUMO2 is an important regulator of endogenous cardioprotection during MIRI and may represent a potential target for future investigation.

## Introduction

Social stress and unhealthy lifestyle have greatly increased the incidence of acute myocardial infarction (AMI) in recent years (Zhao, 2022). During myocardial ischemia-reperfusion injury (MIRI), excessive production of reactive oxygen species (ROS) dysregulates metabolic and inflammatory networks in cardiomyocytes and ultimately culminates in cell death (Hao, 2022). Massive monocytes, macrophages and neutrophils infiltrate and initiate inflammation and adverse cardiac remodeling in the infarcted heart (Liao, 2022). Current clinical interventions mainly restore blood flow into the infarcted heart and save many patients with AMI. However, few therapies are available to eliminate the detrimental effects of ischemia and reperfusion on the cardiac homeostasis and functions (Bei, 2022). Untreated cellular damage and inflammation deteriorate cardiac function, impair cardiac contractility and even trigger a cascade of maladaptive processes towards heart failure (Pastena et al., 2023). Interestingly, post-translational modifications such as SUMOylation appear to play important roles in preventing cardiomyocyte apoptosis and adverse cardiac remodeling (Wang et al., 2023; Fang, 2024; Loh et al., 2023).

SUMOylation describes an important post-translational modification in which small ubiquitin-like modifier (SUMO) proteins, including SUMO1, SUMO2/3, and SUMO4, are covalently attached to target proteins through a multistep enzymatic cascade (Johnson, 2004). SUMO proteins covalently conjugate to specific lysine residues in the consensus sequence ψ-K-X-D/E− and thereby regulate protein activity, intracellular translocation, transcription, DNA repair, and chromatin assembly (Zhao, 2014; Cubenas-Potts and Matunis, 2013; Geiss-Friedlander and Melchior, 2007). SUMO proteins are highly homologous, SUMO1 shares approximately 45% similarity with SUMO2/3, whereas SUMO2 and SUMO3 shares approximately 95% similarity, allowing these proteins to exhibit distinct or overlapping functions *in vivo* (Johnson, 2004; Hecker et al., 2006; Saitoh and Hinchey, 2000). For example, SUMO1 prefers to target RanGAP1 and Nkx2.5 while SUMO2/3 modifies nuclear actin (Saitoh and Hinchey, 2000; Wang et al., 2008; Hofmann, 2009). Unlike SUMO1, SUMO2 and SUMO-3 contain the ψ-**K**-X-E motif which allows the poly-SUMO chain formation (Johnson and Gupta, 2001). Moreover, serine/threonine phosphorylation enhances SUMO-interacting activity towards SUMO1 over SUMO2/3^17^. However, the biological effects of SUMOylation are highly context dependent, and the functional significance of individual SUMO isoforms may vary across cell types and disease settings (Vertegaal, 2022).

SUMO2 and related SUMOylation have recently emerged to be a new cardioprotective mechanism against MIRI (Zhao et al., 2021a). Indeed, we recently demonstrated that naturally occurring isoflavone puerarin upregulated SUMO2, increased protein SUMOylation, reduced myocardial injury and attenuated cardiac remodeling following AMI (Zhao et al., 2021b; Zhao et al., 2022). Consistently, another study also found that SUMO2 protected the heart against MIRI via reducing ROS generation and inhibiting apoptosis (Gu, 2014). These results suggest that SUMO2 and related SUMOylation may represent therapeutical targets for MIRI.

In this study, we aimed to validate the cardioprotective role of SUMO2 in myocardial ischemia-reperfusion injury. We generated heart specific SUMO2 knockout (SUMO2-KO) mice, determined the effects of SUMO2 expression on myocardial injury and mice and attempted to gain the mechanistic insights.

## Materials and methods

1. Antibodies and Reagents

Dulbecco’s modified Eagle’s medium (DMEM), fetal bovine serum, penicillin, and streptomycin were purchased from Thermo Fisher Scientific (Waltham, MA, USA). Antibodies against SUMO2/3, Bax, Bcl-2 and Alexa Fluor 488/647-conjugated goat anti-IgG secondary antibodies were purchased from Invitrogen (Carlsbad, CA, USA). Anti-caspase-3 and anti-p53 antibody were purchased from Santa Cruz Biotechnology Inc (Dallas, Texas, USA), whereas antibody against β-actin was purchased from Cell Signaling Technology (Boston, MA, USA). ML-792 was purchased from Selleck Chemicals (Houston, TX, USA). Pifithrin-a hydrobromide was purchased from MCE (Pudong, SH, CN). Unless otherwise indicated, other biochemical reagents were purchased from Sigma-Aldrich (St. Louis, MO, USA).

2. H9c2 cells culture and oxygen-glucose deprivation/reoxygenation (OGD/R)

Rat embryonic cardiomyocyte H9c2 cells were obtained from the American Type Culture Collection (Manassas, VA, USA) and cultured in high-glucose DMEM containing 10% FBS, 100 U/mL penicillin, and 100 μg/mL streptomycin at 37 °C in a humidified incubator containing 5% CO_2_. For OGD/R treatment, H9c2 cells were washed twice with PBS and preincubated in glucose-free DMEM with or without compounds treatment, transferred to an Eppendorf Galaxy 48R hypoxia chamber (Hamburg, Germany), and exposed to the conditions of 0.1% O_2_, 5% CO_2_ at 37 °C for 3 h. For reoxygenation, the glucose-free DMEM was replaced with high-glucose DMEM containing 10% FBS. The cells were transferred to the regular cell culture incubator under the conditions of 5% CO_2_ and 95% air for 24 h. For drug treatment, the cells were grown to 70%–80% confluence in the complete growth medium and treated with ML-792 (1 μM), whereas the control cells were treated with an equal amount of dimethyl sulfoxide (DMSO) under the same conditions.

3. Animal husbandry and generation of heart-specific SUMO2-KO mice

The protocols for animal experiments (CULATR 5839–21) were approved by the Committee on the Use of Live Animals in Teaching and Research at the University of Hong Kong. Adult male C57BL/6J mice (10–12 weeks, 25–30 g) were provided by the Centre for Comparative Medicine Research at the University of Hong Kong. Mice were housed in a standard laboratory environment with 12-h light-dark cycle and free access to standard diet and water.

Heart specific SUMO2-KO mice were generated as previously described (Yu, 2020; Agah, 1997). In practice, SUMO2-flox C57BL/6J mice were backcrossed onto a C57BL/6J background from the Jackson Laboratory (Bar Harbor, ME, USA) for 10 generations. SUMO2^f/f^ mice were crossed with Myh6-Cre mice (JAX stock#011038, C57BL/6 background) to generate SUMO2^f/+^; Myh6-Cre mice. The SUMO2^f/+^; Myh6-Cre mice were crossed with homozygous SUMO2^f/f^ mice to generate experimental animals as follows: SUMO2^f/f^; Myh6-Cre (SUMO2-KO) mice and control mice (SUMO2^f/f^ or SUMO2^f/+^). The animals were subjected to PCR-based genotyping with ear genomic DNA and specific primers (Myh6-Cre-F: ATGACAGACAGATCCCTCCTATCTCC, and Myh6-Cre-R: CTCATCACTCGTTGCATCATCGAC). The deletion of SUMO2 exon 4 was verified by PCR detection of heart genomic DNAs with primers: Myh6-Cre-internal-F: CAAATGTTGCTTGTCTGGTG, Myh6-Cre-internal-R: GTCAGTCGAGTGCACAGTTT, SUMO2-genotype-F1: CAATCACAGAGGTGAGCCACT, SUMO2-genotype-R1: CAGCTAAGCACTGCATTGGA.

4. MIRI induction in mice

Experimental ischemia-reperfusion injury was induced by ligating the left anterior descending (LAD) coronary artery as previously described (Zhao et al., 2021b). In brief, mice were anesthetized by intraperitoneal (i.p.) injection of 100 mg/kg ketamine and 10 mg/kg xylazine 15 min before the surgery. A 0.5 cm incision was made on the left chest to expose the heart. By opening the thoracic cavity with a retractor, the LAD coronary artery was ligated from 3 mm below its origin using a 7.0 silk proline suture. The mice were exposed to ischemia for 30 min and subsequent reperfusion for either 24 h or 2 weeks. The mice in the sham group underwent the same procedure except for the ligation step. 4-6 mice in each group were used for each experiment. Mice were imaged by echocardiography prior to tissue harvesting 2 weeks after surgery. The general distress levels of the mice were assessed for determining the appropriate humane endpoint, while the selected ones were euthanized by i.p. injection of overdose pentobarbital (200 mg/kg).

5. Analysis of infarction size by TTC staining

Mouse hearts were sectioned into 1–2 mm-thick pieces and stained with 2,3,5-triphenyl tetrazolium chloride (TTC) following the published procedure (Zhang, 2023). In brief, the five heart sections were incubated in 1% TTC staining solution at 37 °C for 20 min and imaged. The infarct size was analyzed with the ImageJ software (NIH, Bethesda, USA) and normalized to the area at risk (AAR).

6. Histological analysis

The heart specimens were fixed with 4% paraformaldehyde overnight and dehydrated with 30% sucrose for 24 h. Subsequently, the tissues were embedded in optimal cutting temperature compound (OCT) and sectioned into 10 μm-thick slices using a Cryostat from Leica (Leica CM 1950 Cryostat microtome system, Leica Microsystems Limited, Wetzlar, Germany). The sections were subjected to H&E staining, Masson’s trichrome staining and dihydroethidium (DHE) staining.

7. Lentivirus-mediated overexpression or silencing of SUMO2 in H9c2 cells

Lentiviral expression vector plasmids of pCDH-CMV-SUMO2 encoding rat SUMO2-cDNA and pLKO.1-CMV-SUMO2 shRNA with the sequence of 5′- CCGGGACTGAGAACAAC GATCATATCTCGAGATATGATCGTTGTTCTCAGT CTTTTTGAATT-3′ were purchased from IGE Biotechnology Ltd (Guangzhou, China). For the preparation of lentiviral particles, 293 TN cells were pre-seeded, co-transfected with the package of lentivirus packaging vectors (e.g., pRSV-Rev, pMDLg/pRRe, pMD2.G) and plasmid of pCDH-CMV-SUMO2 or pLKO.1-CMV-SUMO2. The transfected cells were cultured at 37 °C for 48 h to allow the production of lentiviral particles. The supernatant was concentrated and purified via ultracentrifugation. For the overexpression and silencing of SUMO2, H9c2 cells were subsequently infected with the corresponding lentiviral particles over 48 h. The stable clones were selected in cell culture medium containing 1 μg/ml puromycin.

8. Western blotting analysis

Proteins from heart tissue or H9c2 cells were extracted and separated by SDS-PAGE for immunoblotting analysis as previously described (Zhao et al., 2022). Total proteins were separated on a 10% polyacrylamide gel and transferred onto 0.22 µm PVDF membrane. The membrane was blocked with 5% non-fat milk solution and incubated with specific primary antibodies at 4 °C overnight. After three washes, the membrane was incubated with HRP-conjugated secondary antibody. The protein levels were detected with Amersham ECL detection reagent (Cytiva, RPN2235) and imaged on ChemiDoc XRS + imaging system (Bio-Rad, California, USA).

9. RNA isolation and quantitative real-time PCR

The mRNA expression of selected biomarkers was measured by qRT-PCR technique as described (Zhang, 2023). Briefly, the total RNAs were isolated from the cells using TRIzol reagent (Invitrogen, CA, USA) and converted to the corresponding cDNAs using RevertAid first-strand cDNA synthesis kit from Thermo Fisher (Waltham, MA, USA). qRT-PCR amplification was performed with specific primers for SUMO2 (Forward: ACGAGAAACCCAAGGAAGGA; Reverse: CTCCATTTCCAACTGTGCAG) from IDT (Coralville, Iowa, USA) and detection reagent SYBR Green mix (Vazyme, Shanghai, China).

10. Assay of serum creatine kinase (CK) and Creatine Kinase MB (CK-MB)

Mouse serum was collected from the heart and measured within 12 h. Serum CK levels were measured using the Creatine Kinase Assay Kit (Cat.BC1145-100T/96S, Solarbio, China) following the manufacturer’s instructions. The activity of CK was determined by measuring the production of molybdenum blue. On the other hand, the serum CK-MB levels were measured using mouse creatine kinase MB isoenzyme Elisa kit (Cat. SEKM-0152-48T, Solarbio, China) following the manufacturer’s instructions. Tetramethyl benzidine (TMB) was used to quantify the activity of bound HRP. The absorbance at 450 nm wavelength was measured to negatively correlate with the CK-MB concentration in the samples.

11. Echocardiography.

Cardiac functions were assessed by echocardiography on VisualSonics Vevo 2100 Imaging System as previously described (Zhang, 2023). Prior to the assessment, mice were anesthetized with 2 L/min Isoflurane. For capturing the cardiac images via the parasternal short axis, mice were exposed to 1 L/min isoflurane via a nose cone. The left ventricular ejection fraction (LVEF) and left ventricular fractional shortening (LVFS) were calculated by using the Vevo 2100 analysis software.

12. Construction and administration of adeno-associated virus serotype 9 (AAV9)

Full length SUMO2 cDNA was inserted into pAAV-cTNT-luciferase vector (Addgene, #69915) at BamHI and EcoRI restriction enzyme sites. pAAV-cTNT-SUMO2 vector, AAV9 packaging plasmid pAAV2/9n (Addgene, #112865) and helper plasmid pAdDeltaF6 (Addgene, #112867) were introduced to HEK293T cells for the preparation of viral particles. The supernatant was concentrated and purified via ultracentrifugation. Viral titers were determined by qPCR technique. Viral genomic titers were quantified relative to standard curve. SUMO2-AAV9 viruses (5 × 10^10^ vg) in a volume of 10 μL PBS were injected into the ischemic heart tissuesWIE. After 2 weeks, all mice were euthanized by injecting overdose pentobarbitone (200 mg/kg, i.p).

13. Flow cytometric analysis

Cell apoptosis was assayed by flow cytometry on Agilent NovoCyte Advanteon BVR. Briefly, following OGD/R treatment, H9c2 cells were collected and double stained with Annexin V-FITC and PI staining solution for 20 min. After incubation, the cells were resuspended gently and analyzed within 1 h. The data analysis was carried out using Flowjo.

14. *In vivo* imaging of AAV-mediated gene expression

Following injection with AAV virus particles, the mouse heart was imaged on IVIS Spectrum system 1 week after MIRI surgery. Briefly, mice were anesthetized by intraperitoneal injection of ketamine (100 mg/kg) and xylazine (10 mg/kg), followed by intraperitoneal administration of luciferin (30 mg/mL in PBS). After 5 min of incubation, the mice were placed in the imaging chamber, and bioluminescent images were acquired using the IVIS Spectrum system.

15. Mitochondrial membrane potential measurement

Changes in mitochondrial membrane potential (MMP) were measured using the JC-1 staining (Aladdin). Briefly, treated cells were washed twice with warm phosphate-buffered saline (PBS). Subsequently, the cells were incubated with 2 µM JC-1 in PBS at 37 °C in the dark for 30 min. As a positive control, 50 µM carbonyl cyanide 3-chlorophenylhydrazone (CCCP), which disrupts mitochondrial integrity and leads to complete loss of MMP, was applied and incubated at 37 °C for another 5 min. Thereafter, the cells were washed three times with pre-warmed PBS and immediately subjected to fluorescence detection using a Nikon fluorescence microscope. When MMP is high, JC-1 spontaneously forms J-aggregates within the mitochondria, emitting red fluorescence. In contrast, when MMP is low, JC-1 diffuses out of the mitochondria and exists as monomers in the cytoplasm, emitting green fluorescence. Therefore, the MMP can be represented by the ratio of red fluorescence intensity to green fluorescence intensity.

16. Co-immunoprecipitation (Co-IP)

H9c2 cells were washed twice with cold PBS and subsequently lysed in RIPA buffer containing protease inhibitor cocktail (Merck, Darmstadt, Germany), phosphatase inhibitor cocktail (MCE, Shanghai, China) and NEM (MCE, Shanghai, China). Cell lysates were then centrifuged at 10,000 g for 10 min at 4 °C. The supernatants were precleared by adding 1 µg of the control IgG and 20 µl of Protein A/G PLUS-agarose suspension (SANTA CRUZ, Dallas, USA). The mixture was incubated on ice for 30 min. Following centrifugation at 2500 rpm for 5 min at 4 °C, the precleared supernatant was mixed with anti-SUMO2/3 antibody or p53 antibody and incubated for 1 h at 4 °C. Then, 20 μL of Protein A/G PLUS-agarose suspension was added to the antibody–lysate mixture and incubated overnight at 4 °C. The immune complexes were collected by centrifuging at 2,500 rpm for 5 min followed by four washes with PBS buffer. The immune complexes were resuspended in 1× SDS buffer. After heating for 5 min at 98 °C, the samples were placed in ice and cooled for use.

To validate the SUMOylation sites of p53, a co-immunoprecipitation (co-IP) assay was performed in 293T cells transiently transfected with either the intact p53 construct or its mutants (K292R, K386R).

17. *In silico* prediction of SUMO2-modified proteins

SUMOylated proteins were collected from published articles (REFs). The target genes were selected for MI-relatedness at the online platforms DisGeNET (https://www.disgenet.org/) and Comparative Toxicogenomics Database (CTD) (http://ctdbase.org/). Protein-protein interactions were then analyzed on the STRING platform (https://string-db.org/). The results were downloaded and visualized by using Cytoscape 3.9.1.

18. Statistics and Reproducibility

Statistical analysis was conducted using One-way or Two-way ANOVA with Prism 8 (GraphPad Prism). A p-value of less than 0.05 was considered statistically significant.

## Results

1. Heart specific SUMO2-KO mice were generated and characterized

**FIGURE 1:**
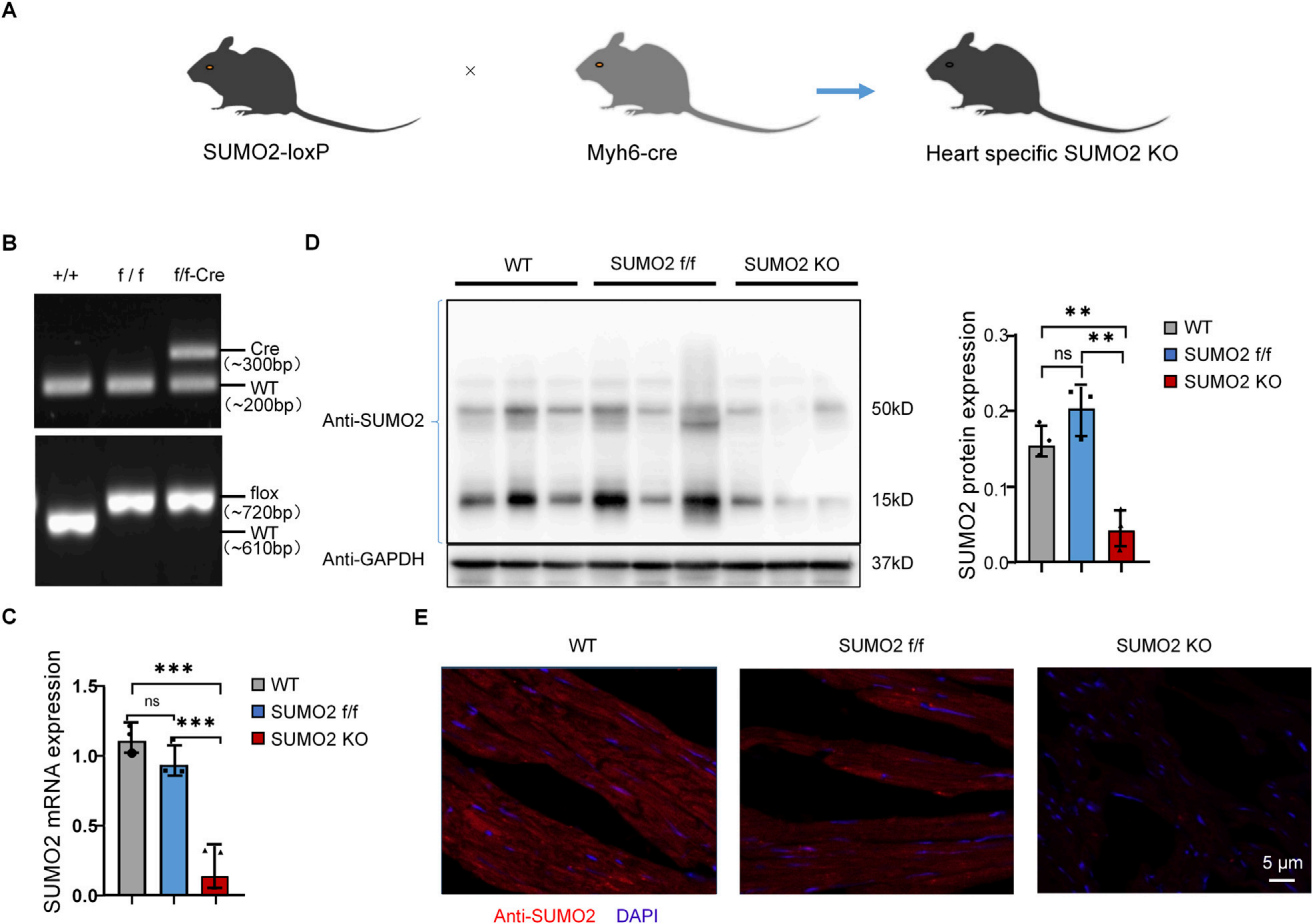
Generation and characterization of heart specific SUMO2-KO mice. **(A)** Cross-mating for the generation of SUMO2-KO mice. **(B)** Verification of deletion of SUMO2 in the hearts with PCR. +/+, wild-type mice; f/f, homozygous for the floxed SUMO2 allele; f/f-Cre, heart-specific SUMO2-KO mice. **(C)** qRT-PCR analysis of SUMO2 mRNA expression in ventricle tissues. **(D)** Western blotting analysis of SUMO2 protein in ventricle tissues. **(E)** Immunofluorescence staining of SUMO2 protein in the heart tissues. WT, wild-type mice; SUMO2 f/f, homozygous for the floxed SUMO2 allele; SUMO2-KO, heart-specific SUMO2-KO mice. Scale bar: 5 μm. Data statistical analysis was conducted using One-way ANOVA, followed by Tukey’s post-hoc test for multiple comparisons and presented as means ± SEM (n = 3/group). *, p < 0.05; **, p < 0.01; ***, p < 0.001; ns means not significant.

To delete SUMO2 in the heart, we crossed SUMO2^f/f^ mice with Myh6-Cre mice to generate SUMO2-KO mice as outlined in Figure 1A. Ear genomic DNAs were extracted for genotyping by PCR technique. As results, PCR products showed a band of 200 bp for the wild-type allele and another band of 300 bp for the Cre allele, suggesting successful Cre knock in. Compared with SUMO2^f/f^ or SUMO2^f/+^ littermates, SUMO2-KO mice showed the knock-in signals of Cre and flox (Figure 1B). We further determined the expression levels of SUMO2 mRNA in the heart through qRT-PCR analysis. Figure 1C showed that the levels of SUMO2 mRNA were markedly reduced in SUMO2-KO mice. On the other hand, the expression of SUMO2 protein in the hearts was assessed by Western blotting. Figure 1D showed that SUMO2 levels were reduced by ∼50% of control levels. Moreover, immunofluorescence detection also confirmed a substantial reduction of SUMO2 protein in the myocardial tissues of SUMO2-KO mice (Figure 1E). It should be noted that this partial reduction likely reflects incomplete recombination, and given the high sequence homology between SUMO2 and SUMO3, the observed signals may be influenced by cross-reactivity in our molecular assays. Despite these limitations, our data collectively confirm the successful generation of a cardiac-specific SUMO2 deficiency model for subsequent functional characterization.

2. SUMO2-KO mice were more vulnerable to myocardial injury

**FIGURE 2:**
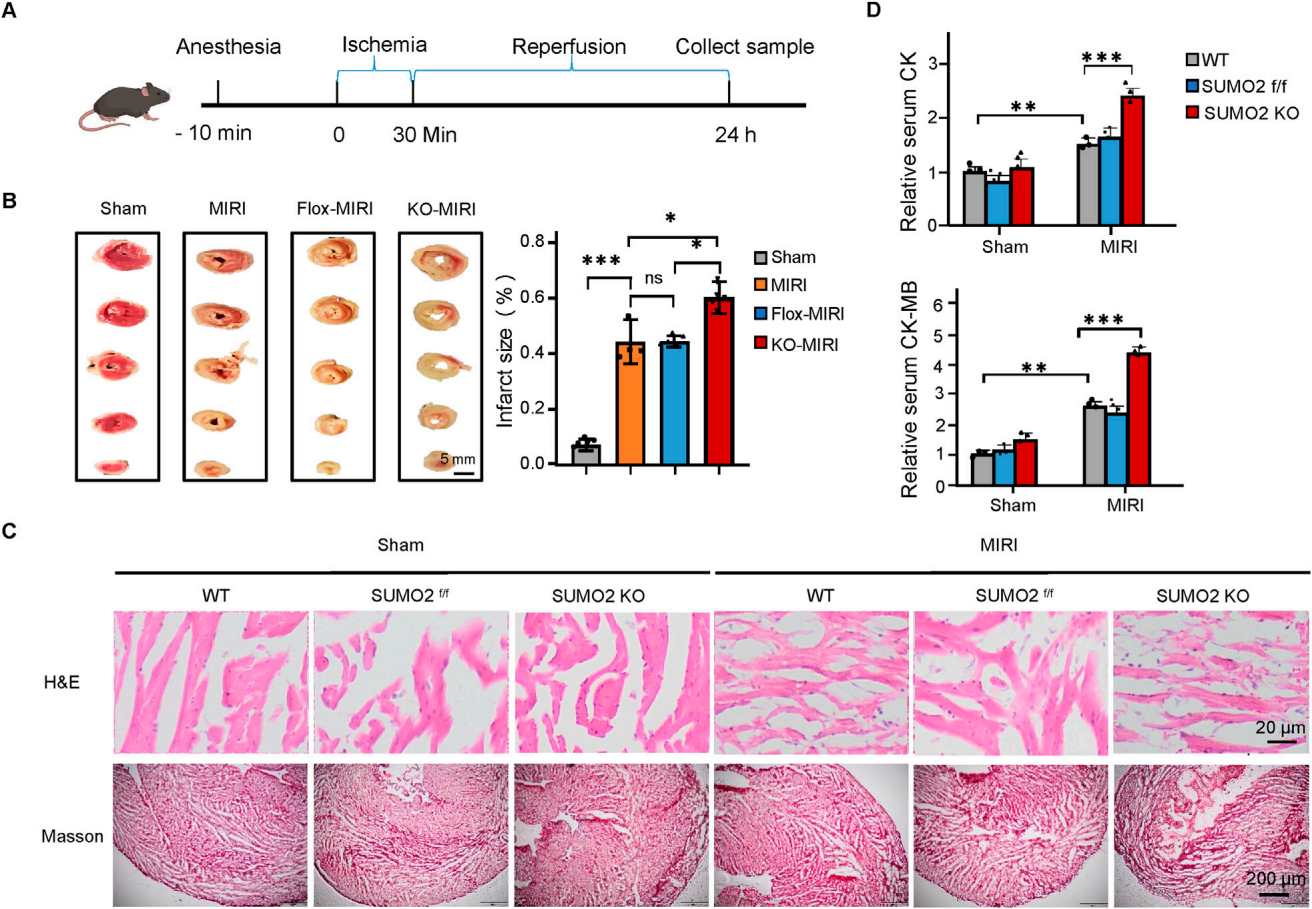
SUMO2 knockout exacerbated myocardial injury in experimental myocardial ischemia-reperfusion injury. **(A)** Experimental scheme for the induction of MIRI in mice. **(B)** TTC staining and quantification of infarct size. Scale bar: 5 mm. **(C)** H&E staining of heart tissues (upper row, scale bar: 20 μm), Masson’s staining of the heart tissue (bottom row, scale bar: 200 μm). **(D)** Serum levels of CK and CK-MB. Data statistical analysis was conducted using two-way ANOVA with Šidák’s multiple comparisons test and presented as means ± SEM (n = 4–6/group). *, p < 0.05; **, p < 0.01; ***, p < 0.001.

To determine the cardioprotective effect of SUMO2, myocardial ischemia-reperfusion injury was induced in WT, SUMO2^f/f^ and SUMO2-KO mice by the LAD ligation procedure as outlined in Figure 2A. After reperfusion for 24 h, mice were euthanized whereas heart tissues and serum samples were collected. As results, TTC staining in Figure 2B showed that infarct size was substantially increased in SUMO2-KO mice. H&E and Masson’s staining in Figure 2C revealed that SUMO2-KO increased the infiltration of immune cells and promoted the disruption of tissue integrity. The blood samples were analyzed for the levels of CK and CK-MB by commercial assay kits. As shown in Figure 2D, MI markedly increased the serum levels of CK and CK-MB, indicating successful induction of MIRI. MIRI caused the leakage of CK and CK-MB into the blood circulation, indicating severe myocardial injury. Taken together, these findings suggested that SUMO2 knockout exacerbated myocardial injury in experimental myocardial ischemia-reperfusion injury.

3. SUMO2 knockout impaired heart functions in post-infarcted mice

**FIGURE 3:**
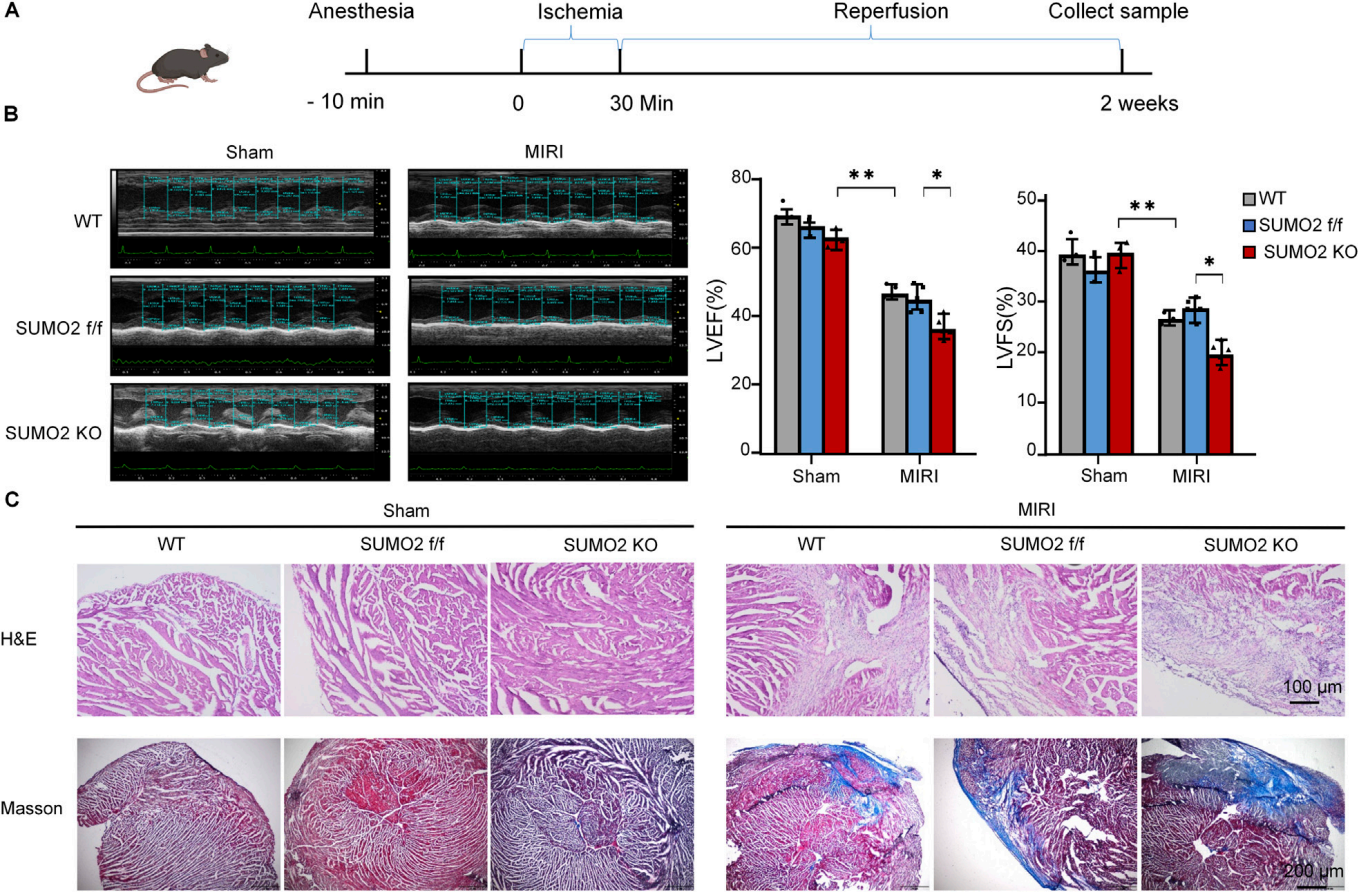
SUMO2 knockout exaggerated the impairment of heart function in MIRI model. **(A)** Experimental scheme for modeling the long-term effects of MIRI in WT, SUMO2^f/f^ and SUMO2-KO mice. **(B)** Echocardiography and quantification of LVEF and LVSF values. **(C)** Histological examination of heart tissues. After the induction of MIRI, mice were subjected to reperfusion for 2 weeks and euthanized. Heart tissues were stained with H&E stain, scale bar: 100 μm, and Masson’s trichrome dyes, scale bar: 200 μm. Data statistical analysis was conducted using two-way ANOVA with Šidák multiple comparisons test and presented as means ± SEM (n = 4–6/group). *, p < 0.05; **, p < 0.01.

To evaluate the effect of SUMO2 on long-term cardiac dysfunction, echocardiography was performed on day 14 after MIRI to assess left ventricular ejection fraction (LVEF) and left ventricular fractional shortening (LVFS), as shown in Figure 3A. MIRI-induced reductions in LVEF and LVFS were more pronounced in SUMO2-KO mice than in control mice (Figure 3B). In addition, H&E staining revealed greater myocardial disarray and inflammatory cell infiltration, while Masson’s trichrome staining showed increased interstitial fibrosis and scar formation in SUMO2-KO mice (Figure 3C). These findings indicate that SUMO2 knockout exacerbates MIRI-induced cardiac dysfunction and structural remodeling over the 14-day period.

4. Ectopic expression of SUMO2 ameliorated cardiac dysfunction against MIRI

**FIGURE 4:**
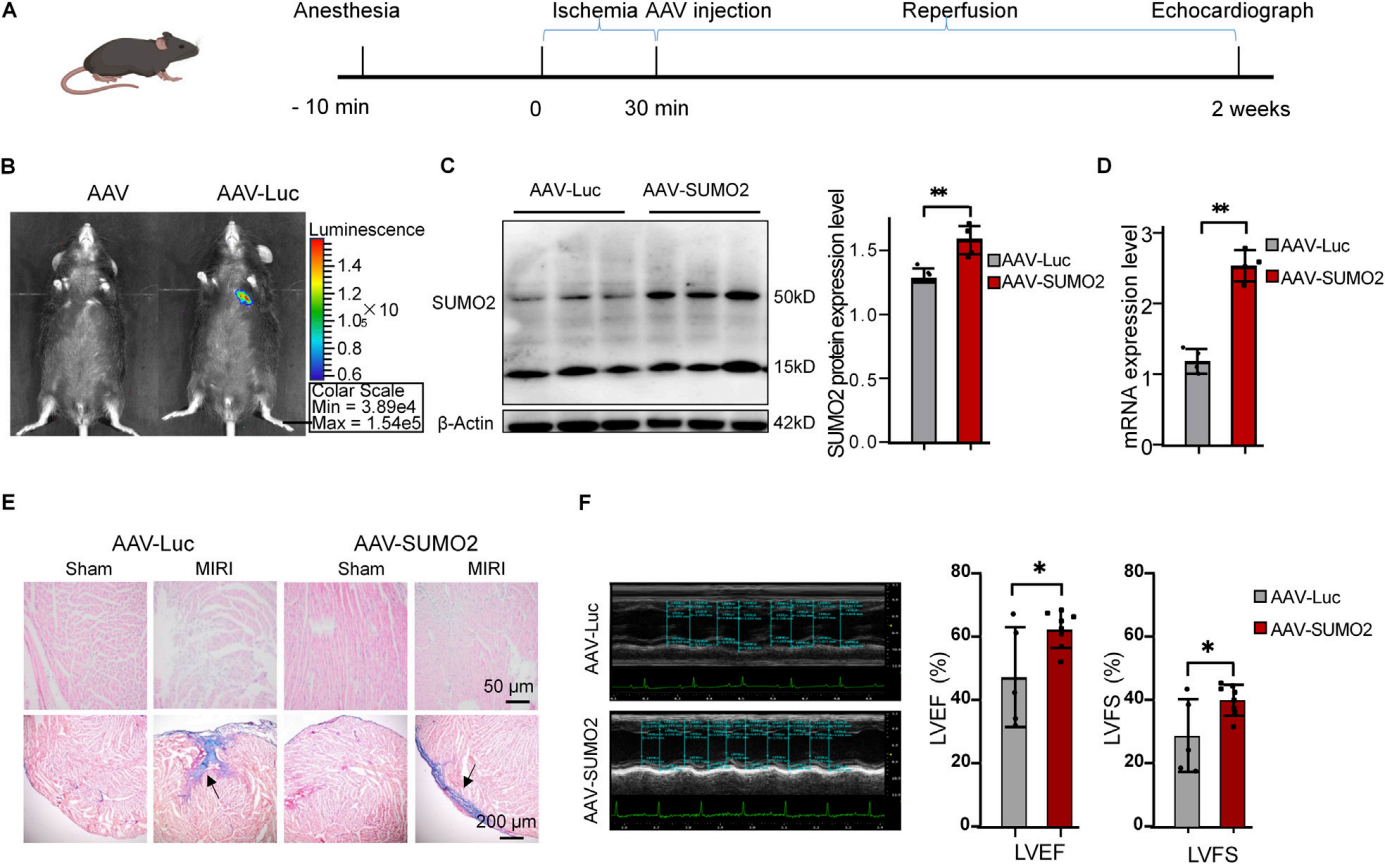
Cardiac SUMO2 transfection supported SUMO2-KO mice against MIRI. **(A)** Cardiac injection of AAV9-Luc or AAV9-SUMO2 viral particles in mice during MIRI. AAV9-Luc (luciferase control) and AAV9-SUMO2 (SUMO2 overexpression). **(B)** IVIS scanning for luciferase expression in the heart, scale bar is 1 cm. **(C)** Western blotting analysis of SUMO2 expression and conjugation (the smears). **(D)** qRT-PCR analysis of SUMO2 expression. **(E)** H&E staining of heart tissues (upper row, scale bar: 50 μm), Masson’s staining of the heart tissue (bottom row, scale bar: 200 μm). **(F)** M mode echocardiography and quantification of LVEF and LVFS values. Data statistical analysis was conducted using one-way ANOVA, followed by Tukey’s post-hoc test for multiple comparisons and presented as means ± SEM (n = 4–6/group). *, p < 0.05; **, p < 0.01.

To restore SUMO2 expression in SUMO2-KO mice, we employed an AAV9 gene delivery system to restore SUMO2 *in vivo* and validated the cardioprotective effect of SUMO2 against MIRI as outlined in Figure 4A. To verify the efficiency of gene transfer, luciferin was intraperitoneally administered to mice. AAV9-mediated luciferase expression was detected in the heart on IVIS imaging system. (Figure 4B). SUMO2 levels in heart tissue were assessed by Western blotting and qRT-PCR techniques. Figures 4C,D showed that cardiac injection of AAV9-SUMO2 virus particles substantially increased the expression of SUMO2 at the protein and mRNA levels. Intriguingly, SUMO2 specific antibody not only detected SUMO2 protein but also showed that SUMO2ylated proteins with larger molecular sizes, predominantly at 50 kD, were markedly increased. H&E and Masson’s staining indicated that SUMO2 transfection effectively protected mice from MIRI-cardiac remodeling (Figure 4E). Echocardiographic examination confirmed that SUMO2 transfection greatly increased LVEF and LVSF and enhanced cardiac performance (Figure 4F).

5. SUMO2 level determined the survival of H9c2 cells against OGD/R injury

**FIGURE 5:**
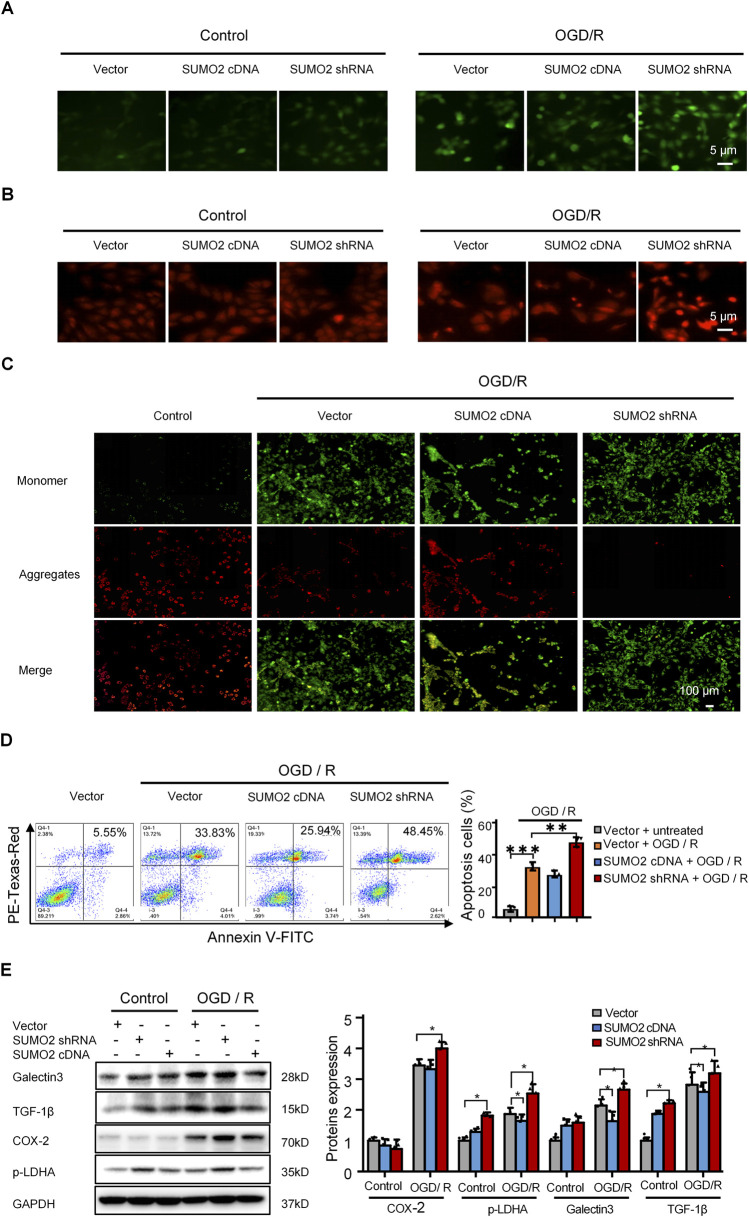
Mechanisms underlying the cardioprotective effects of SUMO2 overexpression in H9c2 cells. **(A)** SUMO2 silence allowed OGD/R-induced production of superoxide anion in mitochondria. ROS generation was detected by DCFH-DA in H9c2 cells (green), Scale bar: 5 μm. **(B)** Mito-SOX probe was used to detect superoxide anion in mitochondria (red); SUMO2 silence allowed OGD/R-induced ROS accumulation in H9c2 cells. **(C)** SUMO2 silence allowed OGD/R-induced disruption of mitochondrial membrane potential. **(D)** SUMO2 silence allowed OGD/R-induced apoptosis in H9c2 cells. **(E)** Western blotting analysis of inflammatory and fibrotic biomarkers. COX2, galectin-3, TGF-β1 and p-LDHA H9c2 cells were analyzed by Western blotting with specific antibodies. Vector (vector control), SUMO2 cDNA (SUMO2 overexpression) and SUMO2 shRNA (SUMO2 silence). Data statistical analysis was conducted using two-way ANOVA with Šidák multiple comparisons test and presented as means ± SEM (n = 3/group). *p < 0.05; **p < 0.01; ***, p < 0.001.

To determine the cardioprotective effects of SUMO2, H9c2 cells were divided into three groups: control, SUMO2-overexpressing, and SUMO2-silenced cells, by transfection with SUMO2-cDNA, SUMO2-shRNA, or the corresponding vector control using a lentiviral gene delivery system. After confirming SUMO2 expression at both the mRNA and protein levels, the stable cell lines were subjected to OGD/R. Oxidative stress was first evaluated by measuring intracellular ROS and superoxide levels using DCF-DA and MitoSOX staining, respectively. As shown in Figures 5A,B, SUMO2 silencing significantly increased ROS and superoxide generation. Mitochondrial function was then assessed by JC-1 staining to determine mitochondrial membrane potential. Figure 5C showed that SUMO2 overexpression tended to preserve mitochondrial membrane potential, whereas SUMO2 silencing promoted its disruption. Cell apoptosis was examined by Annexin V/propidium iodide (PI) co-staining followed by flow cytometry. As shown in Figure 5D, SUMO2 silencing markedly increased apoptosis in OGD/R-treated cells. Finally, inflammatory and fibrotic responses were assessed by immunoblotting for galectin-3, TGF-β1, COX-2, and phosphorylated lactate dehydrogenase A (p-LDHA). SUMO2 silencing clearly increased the expression of these inflammatory and fibrotic markers, whereas SUMO2 overexpression modestly reduced their levels (Figure 5E). Collectively, these results suggest that SUMO2 protects cardiomyocytes against OGD/R injury by suppressing oxidative stress, preserving mitochondrial function, inhibiting apoptosis, and reducing inflammatory and fibrotic responses.

6. SUMO2 protected cardiomyocytes against experimental ischemic injury

**FIGURE 6:**
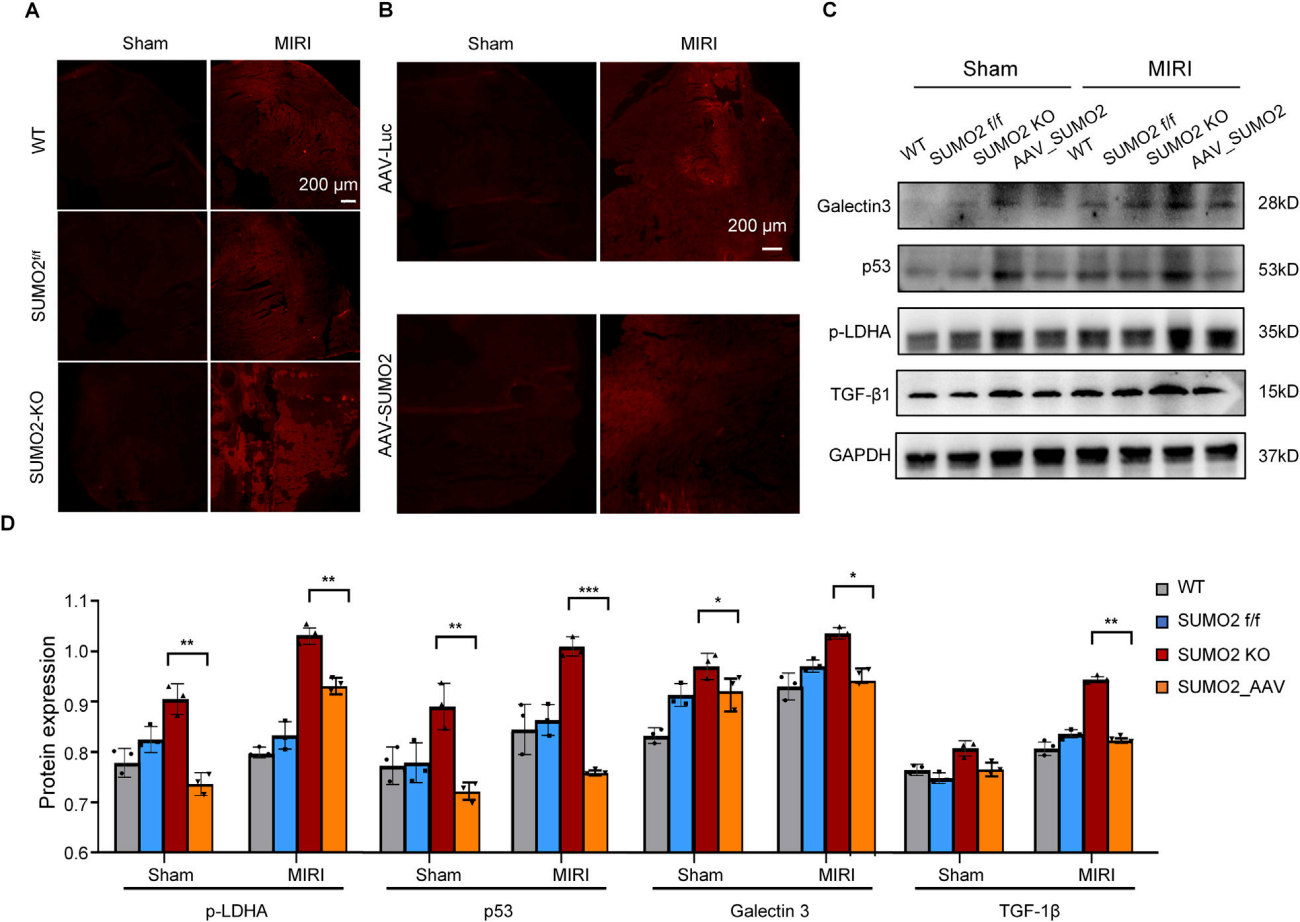
*In vivo* cardioprotective effects of SUMO2 in mice heart tissue. **(A)** SUMO2-KO increased MIRI-induced ROS generation in the risk area of the mice heart. DHE was employed to detect ROS generation in cardiomyocyte of the heart, scale bar: 200 μm. **(B)** SUMO2 overexpression decreased MIRI-induced ROS accumulation. **(C)** Western blotting analysis of biomarkers. Cardiac tissues were collected and analyzed by Western blotting with antibodies against COX2, galectin-3, p53, TGF-1β and pho-LDHA. **(D)** Quantitative analysis of the biomarkers in mouse models of MIRI. Data statistical analysis was conducted using two-way ANOVA with Šidák multiple comparisons test and presented as means ± SEM (n = 3/group). *p < 0.05; **p < 0.01; ***, p < 0.001.

To confirm the mechanisms underlying SUMO2-mediated cardioprotection *in vivo*, heart tissues were collected and made into slices. Mouse heart sections were stained with dihydroethidium (DHE) to assess ROS levels. As shown in Figure 6A, ROS levels were higher in SUMO2-KO mice than in controls. In contrast, SUMO2 overexpression reduced ROS levels in the mouse heart (Figure 6B). On the other hand, adverse cardiac remodeling was assessed by immunoblotting inflammatory and fibrotic biomarkers (e.g., galectin-3, TGF-1β, COX-2, p-LDHA). Figures 6C,D showed that SUMO2 knockout evidently elevated the expression of inflammatory and fibrotic biomarkers whereas SUMO2 overexpression moderately reduced the levels of selected biomarkers. Taken together, these results suggested that SUMO2 might protect cardiomyocytes from MIRI by inhibiting oxidative cell damage, inflammation, and fibrosis.

7. SUMO2 exhibited cardioprotective effects by targeting Bax/Bcl2 signaling pathway

**FIGURE 7:**
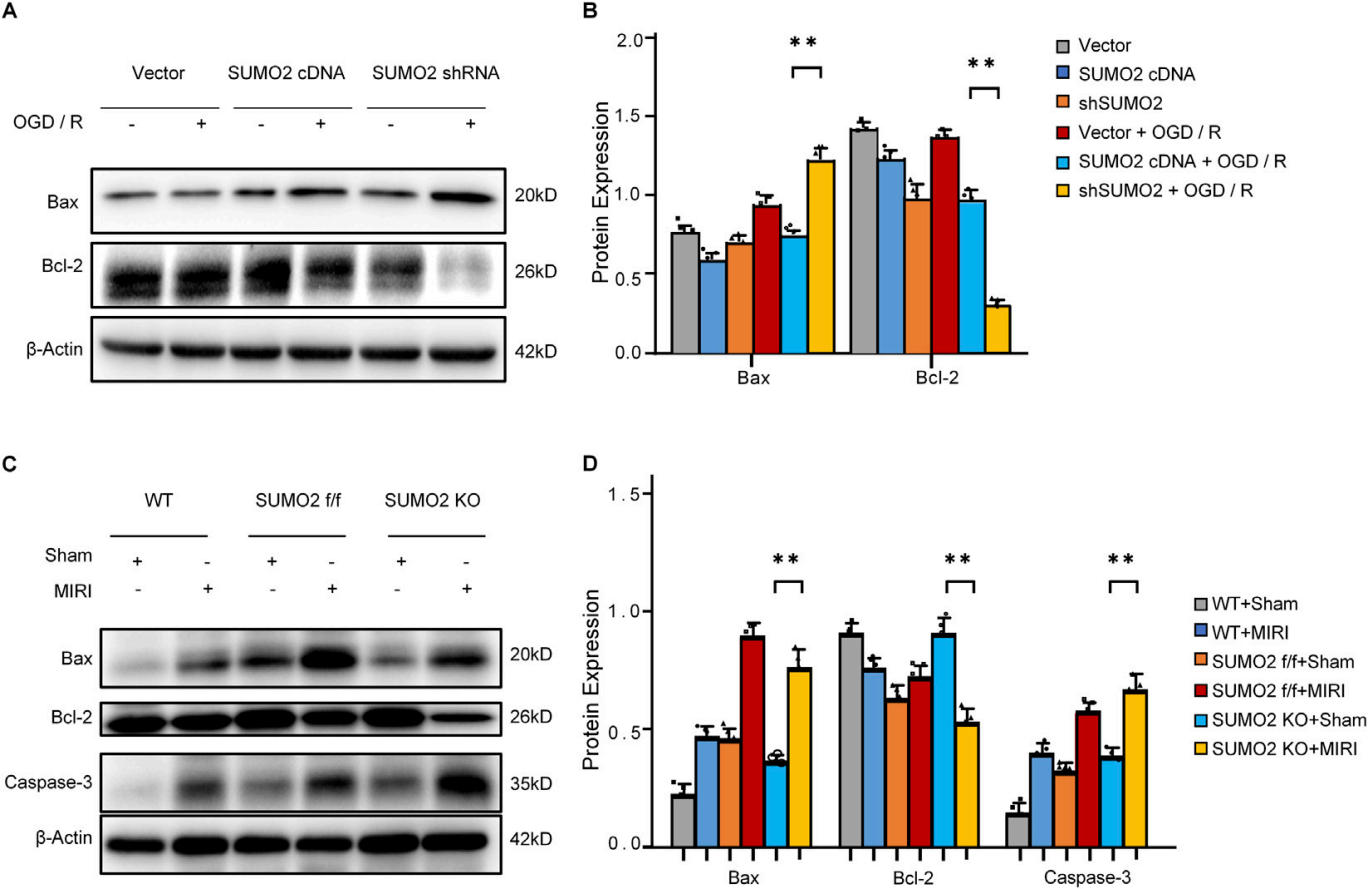
SUMO2 protected cardiomyocytes via regulating Bax/Bcl2 signaling pathway. **(A)** Western blotting analysis of apoptosis biomarkers in H9c2 cells. **(B)** Quantitative analysis of apoptosis biomarkers in H9c2. **(C)** Western blotting analysis of apoptosis biomarkers in mouse models of MIRI. Heart tissues were collected and analyzed by Western blotting with antibodies against Bax, Bcl2 and caspase 3. **(D)** Quantitative analysis of apoptosis biomarkers in mouse models of MIRI. Data statistical analysis was conducted using two-way ANOVA with Šidák multiple comparisons test and presented as means ± SEM (n = 3/group). *, p < 0.05; **, p < 0.01.

Following ROS detection and Annex-V staining for apoptosis, we further determined the levels of apoptosis-associated proteins (e.g., Bax, Bcl-2) in H9c2 cells and mice. After transfection with SUMO2-cDNA, SUMO2-shRNA and vector alone, H9c2 cells were challenged by OGD/R. Indeed, OGD/R increased Bax/Bcl-2 ratio in the vector-transfected cells. Silencing of SUMO2 elevated Bax protein expression while markedly reduced Bcl-2 levels. Conversely, overexpression of SUMO2 marginally decreased the Bax/Bcl-2 ratio (Figures 7A,B). Western blotting analysis in Figures 7C,D basically confirmed that MIRI increased the levels of pro-apoptotic Bax and Caspase-3 while decreased the levels of anti-apoptotic Bcl-2. Importantly, SUMO2 knockout markedly augmented caspase3 expression while decreased Bcl-2 expression during MIRI lesion.

8. SUMO2 may protect cardiomyocytes by involving p53 signaling

**FIGURE 8:**
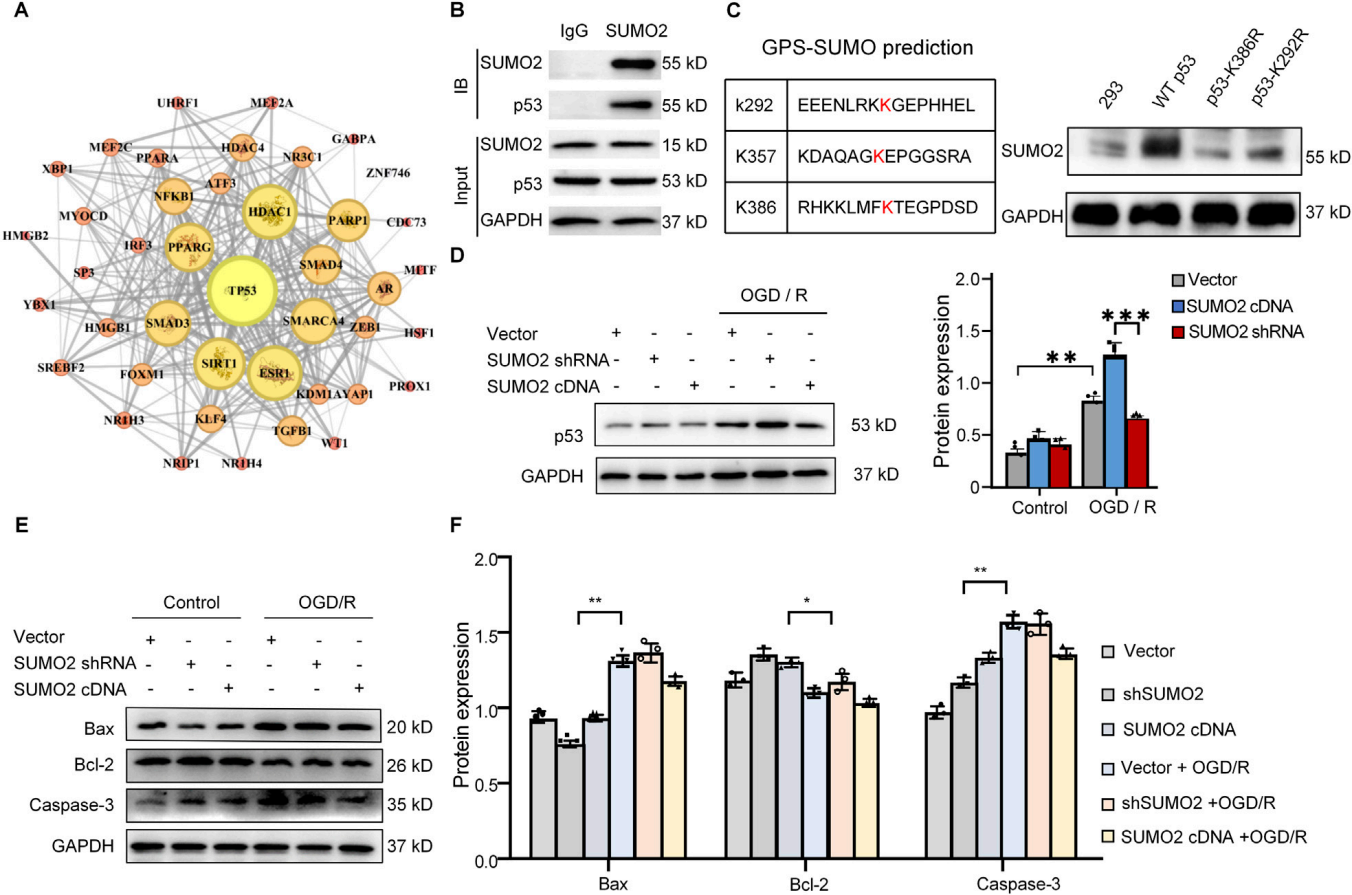
SUMO2 protected cardiomyocytes against ischemic injury through targeting p53. **(A)** Analysis of protein-protein interaction network for key SUMOylated proteins. **(B)** Co-IP validation of SUMO2-p53 interaction. **(C)** Predicted SUMOylation sites of p53(left), Co-IP validation of SUMOylation sites of p53 (right). **(D)** Western blotting analysis for the correlation of p53 and SUMO2. **(E)** Western blotting analysis for the effect of p53 inhibitor on apoptosis biomarkers. **(F)** Quantitative analysis of apoptosis biomarkers. Data statistical analysis was conducted using two-way ANOVA With Šidák’s multiple comparisons test and presented as means ± SEM (n = 3/group). *, p < 0.05; **, p < 0.01; ***, p < 0.001.

To investigate the anti-apoptotic role of SUMO2 in MIRI, we first performed bioinformatic screening to identify potential SUMO2-related targets. SUMOylated proteins were collected from published proteomic datasets, while MIRI-associated proteins were retrieved from the DisGeNET and CTD databases. The overlapping genes were then subjected to protein-protein interaction (PPI) network analysis. As shown in Figure 8A, p53 emerged as one of the key hub genes, suggesting that it may be an important component of the SUMO2-associated response in cardiomyocyte.

Next, we examined the potential association between SUMO2 and p53 and explored putative SUMOylation sites in p53. Co-IP analysis showed that p53 and SUMO2 were detected in the same protein complex (Figure 8B). In addition, GPS-SUMO software predicted K292, K357, and K386 as potential SUMOylation sites in the p53 sequence, with K292 and K386 receiving higher prediction scores (Figure 8C). To further evaluate this prediction, p53 mutants K292R and K386R were generated, and mutation of these lysine residues reduced p53 SUMOylation levels, supporting the possibility that p53 may be a SUMO2-associated substrate.

Finally, we performed functional assays using the p53 inhibitor pifithrin-α hydrobromide to further examine the SUMO2-p53 axis in OGD/R injury. Western blot analysis showed that OGD/R increased p53 expression, while SUMO2 silencing further enhanced p53 levels and SUMO2 overexpression attenuated this effect (Figure 8D). Moreover, pifithrin-α hydrobromide reduced the SUMO2 silencing-induced upregulation of p53 (Figures 8E,F). Together with the changes observed in the Bax/Bcl-2 pathway, these findings suggest that SUMO2 may modulate p53-related signaling and apoptosis during MIRI. However, whether p53 SUMOylation is required for the cardioprotective effects of SUMO2 remains to be fully determined.

## Discussion

Myocardial ischemia-reperfusion injury not only disrupts the structure, composition and function, but also reshapes the inflammatory, metabolic, transcriptomic, and post-translational networks (Zhang, 2022; Frangogiannis, 2018). Among these processes, SUMOylation has emerged as an important regulator of cardiac stress responses (Zhao et al., 2021a). Although SUMO family members share substantial sequence similarity and may partially compensate for one another, accumulating evidence suggests that individual SUMO isoforms can exert distinct biological functions in disease contexts (Saitoh and Hinchey, 2000; Wang, 2014). In particular, SUMO2 appears to be strongly stress responsive and may play a specific role in the myocardial response to ischemic injury (Yu, 2020; Zhao et al., 2021b; Yan, 2022). However, the direct role of SUMO2 and its downstream regulatory mechanisms in myocardial ischemia-reperfusion injury remain poorly understood.

In the present study, we generated a heart-specific SUMO2 knockout mouse model and used it, together with an *in vivo* AAV-mediated rescue approach, to further define the cardioprotective role of SUMO2 in myocardial ischemia-reperfusion injury (MIRI). This experimental design allowed us to assess both loss-of-function and gain-of-function effects in the heart in a physiologically relevant setting. Our findings demonstrate that SUMO2 deficiency aggravates ischemic injury and promotes adverse cardiac remodeling, whereas SUMO2 overexpression mitigates these pathological changes and preserves cardiac function. These results support a protective role for SUMO2 in both the acute and chronic phases of MIRI.

Mechanistically, our data suggest that SUMO2 may regulate cardiomyocyte survival in part through modulation of p53-associated apoptotic signaling. Bioinformatic analysis identified p53 as a potential SUMO2-related target, and this association was supported by co-immunoprecipitation experiments. In both *in vivo* and *in vitro* models, SUMO2 suppression was accompanied by increased p53 expression, an altered Bax/Bcl-2 balance, and enhanced caspase-3 activation, indicating activation of the intrinsic apoptotic pathway. These findings are consistent with the concept that SUMO2 helps limit oxidative stress-induced apoptosis during ischemia-reperfusion. Nevertheless, the present evidence remains largely correlative, and further work is needed to determine whether p53 is directly SUMOylated by SUMO2 and how this modification affects p53 function in cardiomyocytes.

Our findings are also consistent with prior studies showing that SUMOylation participates in cardiac protection under stress conditions (Johnson, 2004; Zhao, 2014; Vertegaal, 2022). During myocardial ischemia-reperfusion injury, prolonged ischemia is known to alter mitochondrial metabolic networks, increase the production of ROS and superoxide ion (Li, 2018) and activates the intrinsic cell apoptosis pathway via the Bax/Bcl-2 signaling pathway (Zhang, 2019). Compared with SUMO1, SUMO2 is more robustly induced by cellular stress, suggesting that it may be particularly important in ischemic injury responses (Chen et al., 2019; Mendler et al., 2016; Chang and Yeh, 2020). Previous studies have implicated SUMO2/3 in regulating oxidative stress, mitochondrial function, ion-channel activity, and inflammatory signaling in the heart (Liu, 2023; Cai, 2021; Kim, 2019). The present study extends these observations by providing direct *in vivo* evidence that cardiac SUMO2 is required for resistance to MIRI. Importantly, the use of a heart-specific knockout model and an AAV-based rescue model strengthens the causal interpretation of our results and highlights the therapeutic relevance of SUMO2 restoration.

This study has several limitations. First, only male mice were used, so sex-specific differences in SUMO2 function during MIRI were not assessed. Second, the *in vitro* experiments were performed in a single cell line, H9c2 cells, which may not fully recapitulate the biology of adult cardiomyocytes. Third, although our data suggest that SUMO2 deficiency is associated with increased oxidative stress and apoptosis, the analyses of these processes are largely descriptive, and the upstream regulatory mechanisms remain to be fully elucidated. In addition, we did not establish animal models carrying site-directed mutations of p53 SUMOylation sites, limiting our ability to confirm a direct mechanistic link between SUMO2-dependent SUMOylation and p53 regulation *in vivo*. Furthermore, as noted in our model validation, the high sequence homology between SUMO2 and SUMO3, together with the possibility of incomplete recombination in the Myh6-Cre model, suggests that some of the observed effects may reflect the combined SUMO2/3 axis or residual SUMO2 activity. Future studies using inducible cardiac-specific models, primary cardiomyocytes, and SUMOylation-deficient p53 mutants will be valuable for addressing these issues.

In conclusion, this study identifies SUMO2 as an endogenous cardioprotective factor in MIRI. By establishing a novel heart-specific SUMO2-KO mouse model and demonstrating the protective effects of *in vivo* AAV-mediated SUMO2 overexpression, we provide evidence that SUMO2 limits oxidative stress, apoptosis, and adverse remodeling after ischemic injury. These findings not only improve our understanding of the molecular basis of MIRI but also suggest that SUMO2 may be a promising therapeutic target for myocardial infarction and related ischemic heart diseases.

## Data Availability

The original contributions presented in the study are included in the article/supplementary material, further inquiries can be directed to the corresponding author.
